# Supplementary material for: Acceptance and compliance with micronutrient powder (MNP) among children aged 6–23 months in northern Nigeria
Source: PLOS Glob Public Health. 2022 Oct 17;2(10):e0000961. doi: 10.1371/journal.pgph.0000961 (PMC10022258; doi:10.1371/journal.pgph.0000961)
Supplement: S1 Dataset — (ZIP) [file pgph.0000961.s008.zip › S8_Dataset/Direct Observations.zip!/Direct Observations/220615_A_GA_AS_1_DO.pdf]

1.1.1 Instrument F –Direct Observation Form

<<Instrument F Direct Observation Form>>

PI Name: PragyaMathema,Stephen Kodish
Study Title: Formative research to develop an infant and young child feeding (IYCF) intervention with micronutrient powder (MNP) for children aged 6 – 23 months
PI Version/Date: Version1.0/January 24, 2015

Date: ___22TH JUNE, 2015_________________Location: _____SANDOKA L.G.A___________________

Data Collector: ___ANTIYA SANDA___________________Participant ID: _A HOUSE BY THE HIGHWAY___

Main goals of this direct observation are to answer the following questions:

1. In this household, what would be the best way(s) to give nutritional supplements to young children? Why?

2. In this household, describe the food sharing that you observe throughout the meal. Are there any predictable patterns?

3. In this household, who is favored and how is this favoritism expressed during meal preparation and eating?

(TEAR OFF AT PERFORATED DOTTED LINE BELOW)

PARTICIPANT ID: _________

Household Composition Table (Fill out as you do data collection)

INITIALS	SEX (M/F)	AGE (estimated)	Relationship to household	Description
ST	F	29 YEARS	WIFE	A SLIM TALL WOMAN
LB	M	9 MONTHS	CHILD	A LITTLE BABY
BY	F	7 YEARS	FEMALE CHILD	A BLACK YOUNG GIRL
TB	F	15 YEARS	OLDEST CHILD	A TALL BLACK GIRL WITH A BLUE WRAPPER
SM	F	2 YEARS	FEMALE CHILD	A SMALL BABY WITH YELLOW PANTS
BS	F	10 YEARS	FEMALE CHILD	A SLIM GIRL WITH A BROWN WRAPPER

PARTICIPANT ID: __________

TIME	ACTOR INITIALS	RECIPIENT INITIALS	WHAT HAPPENED	FOOD	Reactivity
9:00	ST		SWEEPING		
9:05	ST	LB	BATHING HER CHILD		
9:10	ST	LB	BATHING HER CHILD		
9:15	ST	LB	CLEANING HIS BODY WITH TOWEL		
9:20	ST	LB	APPLYING BODY CREAM		
9:25	ST	LB	BREAST FEEDING	BREAST MILK	
9:30	ST	LB	BREAST FEEDING	BREAST MILK	
9:35	ST	LB	WEARING HER CHILD CLOTHES		
9:40	ST	LB	WEARING HER CHILD CLOTHES AND BREAST FEEDING HIM AT THE SAME TIME	BREAST MILK	
9:45	ST	LB	APPLYING POWDER ON HER CHILDS BODY		

TIME	ACTOR INITIALS	RECIPIENT INITIALS	WHAT HAPPENED	FOOD	Reactivity
9:50	ST	LB	WEARING CLOTHE FOR THE CHILD		
9:55	ST		KEEPS HER CHILD ON THE MAT		
10:00	ST		BRINGS TOYS FOR THE CHILD		
10:05	LB		PLAYING ON THE MAT		
10:10	ST	LB	BREAST FEEDING	BREAST MILK	
10:15	ST	LB	BREAST FEEDING	BREAST MILK	
10:16	ST		BRINGS A FLASK AND A CUP		
10:17	ST		POURS KUNU INTO A CUP	GUINEA CORN	
10:18	ST		UNSEAL MNP POWDER		
10:19	ST		POURS MNP INTO KUNU	MNP AND GUINEA CORN	
10:20	ST		MIXED KUNU WITH MNP USING A SPOON	GUINEA CORN	
10:21	ST	LB	CARRYS HER CHILD ON THE LAP		
10:22	ST	LB	STARTS FEEDING HIM WITH KUNU	GUINEA CORN	
10:23	ST	LB	FEEDING HER CHILD WITH KUNU	GUINEA CORN	
10:24	ST	LB	FEEDING HER CHILD WITH KUNU	GUINEA CORN	
10:25	ST	LB	FEEDING HER CHILD WITH KUNU	GUINEA CORN	
10:26	ST	LB	FEEDING HER CHILD WITH KUNU	GUINEA CORN	
10:27	ST	LB	FEEDING HER CHILD WITH KUNU	GUINEA CORN	
10:28	ST	LB	FEEDING HER CHILD WITH KUNU	GUINEA CORN	
10:29	ST	LB	FEEDING HER CHILD WITH KUNU	GUINEA CORN	

TIME	ACTOR INITIALS	RECIPIENT INITIALS	WHAT HAPPENED	FOOD	Reactivity
10:30	ST	LB	FEEDING HER CHILD WITH KUNU	GUINEA CORN	
10:35	ST		KEEPS HER CHILD ON THE MAT		
10:40	ST		TAKES EMPTY CUP TO WASH		
10:45	ST		WASHES THE CUP AND KEEPS IT		
10:50	ST		IS SITTING ON THE MAT		
10:55	LB		PLAYING ON THE MAT		
11:00	LB		PLAYING WITH TOYS ON THE MAT		
11:05	LB		PLAYING ON THE MAT		
11:10	TB		FETCHES WATER IN A POT	WATER	
11:11	TB		IS MAKING FIRE		
11:12	TB		STILL MAKING FIRE		
11:13	TB		BRINGS MORE FIRE WOOD		
11:14	TB		PUTS POT FILLED WITH WATER INSIDE ON THE FIRE	WATER	
11:15	TB		COVERS THE POT WITH WATER INSIDE	WATER	
11:16	TB		IS BLOWING THE FIRE		
11:26	LB		STARTS SLEEPING		
11:31	LB		SLEEPING		
11:36	LB		SLEEPING		
11:41	LB		SLEEPING		
11:46	LB		SLEEPING		
11:51	LB		SLEEPING		

TIME	ACTOR INITIALS	RECIPIENT INITIALS	WHAT HAPPENED	FOOD	Reactivity
11:52	TB		CHECKING THE FIRE		
11:53	TB		BRINGS A BIG BOWL OF MAIZE FLOUR	MAIZE	
11:54	TB		BRINGS A SIEVE AND A BOWL OF MAIZE FLOUR	MAIZE	
11:55	TB		STARTS SIEVING MAIZE FLOUR	MAIZE	
11:56	TB		SIEVING MAIZE FLOUR	MAIZE	
11:57	TB		SIEVING MAIZE FLOUR	MAIZE	
11:58	TB		FETCHES MAIZE FLOUR IN A PAN	MAIZE	
11:59	TB		BRINGS A LONG STICK		
12:00	TB		OPENS THE POT		
12:01	TB		BRINGS MORE FIREWOOD		
12:02	TB		PUTS MORE FIREWOOD INTO THE FIRE		
12:03	TB		BRINGS LONG MIXING STICK		
12:04	TB		RINSES THE LONG MIXING STICK		
12:05	TB		BLOWS THE FIRE		
12:06	TB		IS POURING MAIZE FLOUR INTO BOILING WATER	MAIZE	
12:07	TB		POURING MAIZE FLOUR INTO BOILING WATER ON THE FIRE	MAIZE	
12:08	TB		MIXING MAIZE FLOUR WITH A STICK	MAIZE	
12:09	ST		COMES TO SUPERVISE THE COOKING PROCESS		
12:10	TB		MIXING THE MAIZE IN THE POT WITH STICK	MAIZE	
12:11	TB		POURS LITTLE WATER INTO IT	WATER	
12:12	TB		COVERS THE POT		

TIME	ACTOR INITIALS	RECIPIENT INITIALS	WHAT HAPPENED	FOOD	Reactivity
12:13	TB		PUTS MORE FIREWOOD		
12:14	TB		LEAVES THE KITCHEN		
12:15	TB		GOES TO SIT BUT WATCHING THE FIRE		
12:20	LB		STILL SLEEPING		
12:25	LB		SLEEPING		
12:30	ST		SWEEPING THE COMPUND		
12:35	ST		SWEEPING THE COMPOUND		
12:40	ST		WASHING THE WATER POTS		
12:45	ST		WASHING ALL THE WATER POTS		
12:50	ST		RINSING ALL THE WATER POTS		
12:55	ST		RINSING ALL THE WATER POTS		
1:00	TB		CHECKS FIRE AND ADD MORE FIREWOOD		
1:01	TB		OPENS THE POT TO CHECK IF ITS BOILING		
1:06	LB		WAKES FROM SLEEP		
1:07	TB		OPENS THE POT ON THE FIRE		
1:08	TB		POURS MAIZE FLOUR INTO POT	MAIZE	
1:09	TB		POURS AND MIXES MAIZE WITH A STICK	MAIZE	
1:10	TB		IS MIXING THE MAIZE FASTER	MAIZE	
1:11	TB		IS MIXING THE MAIZE FASTER WITH A STICK	MAIZE	
1:12	TB		CONTINUES TO MIX FASTER	MAIZE	
1:13	TB		POURS MORE MAIZE INTO THE	MAIZE	

TIME	ACTOR INITIALS	RECIPIENT INITIALS	WHAT HAPPENED	FOOD	Reactivity
			POT		
1:14	TB		CONTINUES TO MIX THE MAIZE	MAIZE	
1:15	TB		IS MIXING MAIZE INTO TUWO	MAIZE	
1:16	TB		REMOVES THE STICK		
1:17	TB		COVERS THE POT ON FIRE		
1:18	TB		TAKES THE BOWL OF MAIZE MEAL INTO THE ROOM	MAIZE	
1:19	TB		WASHES THE SERVING PLATE		
1:20	TB		BRINGS THREE PLATE		
1:21	TB		RINSES ALL THE THREE PLATES		
1:22	TB		OPENS THE POT ON THE FIRE		
1:23	TB		BRINGS THE MIXING STICK		
1:24	TB		CONTINUES TO MIX THE TUWO	MAIZE	
1:25	TB		IS MIXING THE TUWO SLOWLY THIS TIME	MAIZE	
1:26	TB		REMOVES THE STICK		
1:27	TB		KEEPS THE STICK ASIDE		
1:28	TB		USES THE SERVING PLATE TO SERVE TUWO	MAIZE	
1:30	TB		REMOVES ALL THE FIREWOOD		
1:31	TB		POURS WATER ON THE FIREWOOD		
1:32	TB		POURS WATER INTO EMPTY TUWO POT		
1:33	TB		COVERS THE POT		
1:34	TB		BRINGS A POT OF SOUP FROM THEIR ROOM	SPINACH LEAVES	
1:35	TB		OPENS THE POT OF SOUP	SPINACH AND GROUNDNUT PASTE	

TIME	ACTOR INITIALS	RECIPIENT INITIALS	WHAT HAPPENED	FOOD	Reactivity
1:36	TB		STARTS SERVING THE SOUP	SPINACH AND GROUNDNUT PASTE	
1:37	TB		SERVES THE SOUP INTO 3 PLATES	SPINACH AND GROUNDNUT PASTE	
1:38	TB		STOPS SERVING THE SOUP	SPINACH AND GROUNDNUT PASTE	
1:39	BY		COMES TO PACK ALL THE PLATES		
1:40	BY		COVERS EACH PLATE FIRST		
1:41	BY		TAKES EACH PLATE TO A ROOM		
1:42	BY		BRINGS A PLATE OF TUWO	MAIZE	
1:43	BY		KEEPS THE PLATE OF TUWO ON THE MAT	MAIZE	
1:44	BY		KEEPS PLATE OF SOUP ON THE MAT	SPINACH LEAVES	
1:45	BY, TB, SM, BS		ALL EATING TOGETHER	MAIZE WITH SPINACH LEAVES	
1:50	BY, TB, SM, BS		ALL EATING FROM THE SAME PLATE	MAIZE AND SPINACH LEAVES	
1:55	ST	LB	BREASTFEEDING	BREAST MILK	
2:00	ST	LB	BREASTFEEDING	BREAST MILK	
2:05	LB		PLAYING ON THE MAT		
2:10	ST	LB	CARRY'S HER CHILD ON THE LAP		
2:15	SB	LB	CARRY'S HER CHILD ON THE LAP		
2:20	SB	LB	CARRY'S HER CHILD ON THE LAP		
2:25	SB	LB	BREASTFEEDING HER CHILD	BREAST MILK	
2:30	SB	LB	BREASTFEEDING HER CHILD	BREAST MILK	
2:35	SB	LB	BREASTFEEDING HER CHILD	BREAST MILK	
2:40	SB	LB	BREASTFEEDING HER CHILD	BREAST MILK	
2:45	SB		KEEPS HER CHILD ON THE MAT		
2:50	LB		STARTS PLAYING		
2:55	LB		PLAYING		
3:00	LB		PLAYING ON THE MAT		
3:05	SB	LB	CARRY'S HER CHILD ON THE LAP		

TIME	ACTOR INITIALS	RECIPIENT INITIALS	WHAT HAPPENED	FOOD	Reactivity
3:10	SB	LB	PLAYING WITH HER CHILD		
3:15	SB	LB	PLAYING WITH HER CHILD		
3:20	SB	LB	STARTS BREASTFEEDING	BREAST MILK	
3:25	SB	LB	BREASTFEEDING	BREAST MILK	
3:30	SB	LB	BREASTFEEDING	BREAST MILK	
3:35	SB	LB	LYING TOGETHER ON THE MAT		
3:40	SB		TRYING TO PUT HER CHILD TO SLEEP		
3:45	SB		SINGING TO HER CHILD		
3:50	LB		STARTS SLEEPING		
3:55	LB		SLEEPING		
4:00	LB		SLEEPING		
4:05	LB		SLEEPING		
4:10	LB		SLEEPING		
4:15	SB, LB		SLEEPING TOGETHER ON THE MAT		
4:20	SB, LB		SLEEPING TOGETHER ON THE MAT		
4:25	SB, LB		SLEEPING TOGETHER ON THE MAT		
4:30	SB		WAKES UP AND GOES TO THE BATHROOM		
4:35	LB		SLEEPING		
4:40	SB		FETCHING WATER FROM WATER POT		
4:45	SB		TAKES A BUCKET OF WATER OUTSIDE		
4:50	SB		CARRY’S HER CHILD ON THE BACK		
4:55	SB		CARRY’S HER CHILD ON THE BACK		
5:00	SB		DROPS HER CHILD ON THE MAT		
5:05	SB		STARTS SWEEPING		
5:10	LB		PLAYING ON THE MAT		
5:15	LB		PLAYING ON THE MAT		
5:20	LB		STARTS CRYING		
5:25	SB	LB	BREASTFEEDING	BREAST MILK	
5:30	SB	LB	BREASTFEEDING	BREAST MILK	
5:35	SB	LB	BREASTFEEDING	BREAST MILK	
5:40	SB	LB	BREASTFEEDING	BREAST MILK	
5:45	SB		KEEPS HER CHILD ON THE MAT		
5:50	SB		STARTS WASHING SOME PLATES		

TIME	ACTOR INITIALS	RECIPIENT INITIALS	WHAT HAPPENED	FOOD	Reactivity
5:55	LB		PLAYING ON THE MAT		
6:00	LB		PLAYING ON THE MAT		
6:05	BY, LB		PLAYING TOGETHER		

PARTICIPANT ID: _________

Primary Questions (Complete after you complete data collection):

From what you have seen observing THIS HOUSEHOLD, please answer the following questions in detail:

Question	Write your detailed response here
1. In this household, what would be the best way(s) to give nutritional supplements such as an MNP to young children to ensure compliance?<br><br>Why?	The best way to give mnp to young children should be in the morning when the child is hungry and ready to eat.<br><br>Because semi-solid foods are prepared in the morning. The young children would comply because mnp would be unnoticeable in the kunu and the young children will drink all.
2. In this household, who is favored during meal preparation or feeding?	The young children are favoured during meal preparation.

How is this favoritism expressed?	They are served first and foremost before anyone else and they are served a huge portion of food on a large plate
3. How could we promote the MNP supplement for this family that would attract their attention?<br><br>Who would they listen to?	The mnp should be promoted through the oldest daugther and the mother. That way, their attention would be attracted.<br><br>They would listen to either the oldest daugther or the mother because they stay at home all day and both of them are the once that make decisions relating to meal preparation and decisions relating to the up keep of the household in general.
4. In this household, what would be the best ways to promote the MNP but limit how much it would be shared?<br><br>Why?	In this household, the best way to promote mnp is to instruct the mother on how to administer the mnp propely. She should be told that the product is meant for her young chid who is 9 months.<br><br>Because other children from the neighbours houses kept coming into the house to pick things. Some sit down to eat together with the children of this household. If not properly instructed, the

	mother or the oldest childcould share the product among the neigbours children or other children could easily access the place where the mnp is kept.
5. What other issues and concerns did you see with this family that are important for us to consider with regard to introduction of an MNP?<br><br>Why?	This household foods ranging from groundnut, guinea corn, millet and soya beansbut do not know that it is important to use them in preparing meals. These foods are left in bays but not used to prepare meals.<br><br>The mother prepared plain guinea corn kunu without adding groundnut paste into it for the young child. When asked, she said she doesn't know that she is supposed to use the groundnut paste in making kunu for the young child.
6. Based on your experience observing this child today, do you believe reactivity negatively impacted your ability to see the food-related behaviors of the child?<br><br>Please explain your answer in detail.	There wasn't any form of reactivity to an extent.<br><br>The mother was playing with the child too often and checking to know if i was watching. In general that did not affect my observation in any way.
